# A Novel Family of Terminal-Repeat Retrotransposon in Miniature (TRIM) in the Genome of the Red Harvester Ant, *Pogonomyrmex barbatus*

**DOI:** 10.1371/journal.pone.0053401

**Published:** 2012-12-28

**Authors:** Yihong Zhou, Sara Helms Cahan

**Affiliations:** Department of Biology, University of Vermont, Burlington, Vermont, United States of America; University of Poitiers, France

## Abstract

We report the first described non-plant family of TRIMs (terminal-repeat retrotransposons in miniature), which are small nonautonomous LTR retrotransposons, from the whole-genome sequence of the red harvester ant, *Pogonomyrmex barbatus* (Hymenoptera: Myrmicinae). Members of this retrotransposon family, named PbTRIM, have typical features of plant TRIMs in length and structure, although they share no overall sequence similarity. PbTRIM elements and their solo-LTRs are abundant in the host genome and exhibit an uneven distribution pattern. Elements are preferentially inserted into TA-rich regions with ATAT as the most common pattern of target site duplication (TSD). PbTRIM is most likely mobile as indicated by the young age of many complete elements, the high degree of sequence similarity among elements at different genomic locations, the abundance of elements in the host genome, and the presence of 4-bp target site duplications (TSDs) flanking the elements and solo-LTRs. Many PbTRIM elements and their solo-LTRs are located within or near genes, suggesting their potential roles in restructuring the host genes and genome. Database search, PCR and sequencing analysis revealed the presence of homologous PbTRIM elements in other ant species. The high sequence similarity between elements from distantly related ant species, the incongruence between the phylogenies of PbTRIM and its hosts, and the patchy distribution of the retroelement within the Myrmicinae subfamily indicate possible horizontal transfer events of the retroelement.

## Introduction

Transposable elements (TEs) are mobile genetic sequences that have been found in most eukaryotic genomes. They account for almost 50% in the human genome [1] and up to 85% in some plant genomes [2]. Traditionally, TEs can be divided into two major classes based on whether their transposition intermediate is RNA (class I) or DNA (class II). The class I TEs, or retrotransposons, translocate via a “copy and paste” mechanism. The element-encoded transcript (mRNA) forms the transposition intermediate, which is reverse-transcribed into cDNA by a TE encoded reverse transcriptase and integrated into a new site of the host genome. In contrast, elements of the class II or DNA transposons normally excise as double-stranded DNA and reinsert elsewhere in the host genome without RNA intermediate [3]–[4].

Class I retroelements can be divided into two major subclasses, long terminal repeat (LTR) retrotransposons and non-LTR retrotransposons, on the basis of transposition mechanism and structure. LTR retrotransposons have two characteristic LTRs in direct orientation that flank the internal region. The sizes of LTR retrotransposons range from a few hundred base pairs up to 25 kilobases, with the LTRs range in size from ∼100 base pairs to several kilobases [5]–[6]. In autonomous (functional) LTR retrotransposons, the internal region contains at least two genes (*gag* and *pol* genes) that encode proteins necessary for retrotransposition. Nonautonomous LTR retrotransposons lack the internal coding domain and are mobilized by proteins provided from functional LTR retroelements (their autonomous counterparts). LTR retrotransposons are predominant in plants but are less abundant in animals. LTR retroelements are important sources of genetic and phenotypic diversity, and have been found to be the major determinant of the tremendous variation in plant genome size [4]. They are also involved in maintaining heterochromatic silence and genomic stability in plants [5], [7]–[8].

To date there are two groups of non-autonomous LTR retrotransposons that have been reported only from plants: the large retrotransposon derivatives (LARDs) and the terminal repeat retrotransposons in miniature (TRIMs). Elements of the two groups are similar in having the typical features of LTR retrotransposons and lacking protein-coding capability in their internal domain. However, they are apparently different in size. LARDs typically have ∼4.4-kb LTRs and ∼3.5-kb central domains [9], whereas TRIMs are less than 1000 bp in size, with the LTRs ranging from ∼100 to ∼350 bp [10]–[11]. TRIM elements are mobile and involved actively in the restructuring of plant genomes, affecting the promoter, coding region and intron-exon structure of genes [10], [12]. Some unique features of TRIM elements, including their small size, insertion preference in euchromatic regions, high copy numbers and relatively even distribution throughout plant genomes, have also made them promising DNA markers for molecular and genetic applications [13]–[14]. TRIMs are conserved in sequence and widespread in all vascular plants investigated to date [11], thus providing a unique resource for the investigation of gene and genome evolution mediated by TEs.

Although TRIM elements are ubiquitous in plants, they have not been found outside the plant kingdom. Here we report a new group of TRIM elements that have been identified in the genome of the red harvester ant, *Pogonomyrmex barbatus*. Ants of the genus *Pogonomyrmex* have been intensively studied for their behavior, ecology and social organization [15]. The discovery of a unique system of genetic queen-worker caste determination in some populations of *P. barbatus*, a contrast to most systems of environmental caste determination in social insects, has also made the ant a model for studying reproductive division of labor [16]–[17]. TEs are one of the principal driving forces behind the evolution of eukaryotic genomes [18]; however little is known about transposons in ants. The recently available genomic sequences of *P. barbatus* [19], as well as several other ant species [20] has provided new opportunities to investigate the composition and distribution of TEs in the ant genome. We annotated retrotransposons in the *P. barbatus* genome and identified a new family of LTR retrotransposon which we refer to as PbTRIM. Based on their small size and non-coding internal domain, PbTRIM can be classified as TRIM, although they have no overall sequence similarity with previously reported plant TRIMs. We analyzed the insertion time, genome distribution pattern, insertion site of PbTRIM elements in the *P. barbatus* genome, and examined the distribution and evolution of the family in other ant species.

## Results

### Annotation of LTR retrotransposons and identification of PbTRIM family

**Figure 1:**
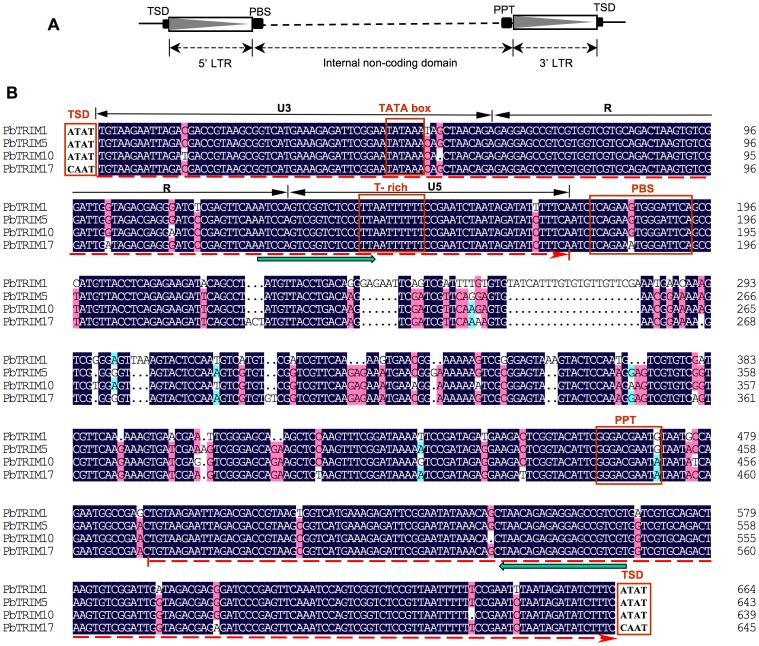
General structure of PbTRIM elements. (A) Schematic diagram of the general structure of PbTRIM elements. (B) Multiple alignment of selected PbTRIM elements from *P. barbatus* genome shows the typical structure of LTR retrotransposons. The 5′ and 3′ LTR are indicated by the leftwards dashed arrows underneath the sequence alignment. Structural features are marked on the top of the aligned sequences. Green thick arrows indicate the PCR primer biding sites. LTR: long terminal repeat; PBS: primer binding site; PPT: polypurine tract; TSD: target site duplicate; U3: unique 3′ RNA region; R: repeated RNA region; U5: unique 5′ RNA region.

Our initial screening of the latest *P. barbatus* genome assembly (version 3) using LTR_FINDER program identified 139 candidate LTR retrotranposons. After manual sequence inspection and BLASTN searches against the GenBank nr database, 132 sequences were annotated as LTR retrotransposons. These LTR retrotransposons can be grouped into 29 LTR retrotransposon families based on sequence similarity (Zhou *et al.*, unpublished data). Notably, out of all the identified LTR retrotransposon elements, ∼30% (40/132) belonged to a single family. Members of the family range in size from 480 to 781 bp and carry LTRs of 152–184 bp. Complete elements are flanked by a 4-bp target site duplication (TSD), the direct short repeat normally generated after insertion of the retrotransposons. The internal domains of the elements lack coding capability. Within the internal region, a highly conserved ∼15-nt primer biding site (PBS), which is complementary to the leucine tRNA of *Drosophila melanogaster*, can be identified immediately downstream of the 5′ LTR, and a ∼15-nt polypurine tract (PPT) is located upstream of the 3′ LTR (Figure 1A, 1B).

Detailed analysis of the LTR sequences of the elements revealed the typical structural characteristics of LTR retrotransposons. LTRs of retrotransposons harbor important regulatory signals for reverse transcription, including sequences involved in the promotion, enhancement and termination of RNA transcription. The LTRs generally contain conserved termini with a 5′- TG … CA-3′ structure, and can be divided into three regions: the unique 3′ RNA (U3), repeated RNA (R) and unique 5′ RNA (U5) regions [5], [21]. The U3 region of PbTRIM elements ranges in size from 61 to 64 bp with the typical TATA box (5′-TATAAA-3′) as the promoter sequence. The R domain is usually 11-bp downstream of the TATA box, starting with G and ending with CA. The putative R domain of PbTRIM elements is 68–70 bp in length. The U5 region typically contains GT or T-rich sequences located within the first 40-bp of the domain, and a TTGT motif, the terminal signal of RNA synthesis. We found T-rich sequence but not TTGT motif in this region (Figure 1B).

This new group of elements has typical features of LTR retrotransposons. BLASTN searches against the nr and genome database in GenBank as well as the comprehensive transposon database, Repbase (http://www.girinst.org), did not detect strong sequence similarity (80% or more sequence identity in at least 80% of their coding or internal domain, or within their terminal repeat regions, or in both; see [3]) with any reported LTR retrotransposon. Meanwhile, no significant TBLASTX match (E-value = 1e-4 or less) was found in those databases using either the complete or internal sequences of the elements as queries. Thus, this group of elements can be considered as a new non-autonomous LTR retrotransposon family which has been named PbTRIM. Although members of PbTRIM show no sequence similarity with plant TRIMs, they have small size and a non-coding internal domain containing PBS and PPT motifs, which are the unique features of TRIMs. Therefore, PbTRIM can be classified as a new family of TRIM.

### Genomic distribution and insertion site preference of PbTRIM elements

To investigate the abundance and genomic distribution of the PbTRIM family, we used the 40 initially identified complete elements as queries in BLASTN searches (E-value = 1e-10 or less) against the *P. barbatus* genome sequences. A total of 270 significant matches were found, including 67 complete elements (27 of them are newly-identified intact sequences that were missed by the LTR_FINDER program [22]), 44 truncated elements and 159 solo-LTRs (a solo-LTR is a single LTR sequence in the genome resulting from unequal homologous recombination between the two LTRs of a retrotransposon). The complete elements are highly conserved in their LTR regions, but are more variable in their internal regions. They generally share 81–99% sequence similarity with each other (see File S1 for the multiple sequence alignment).

**Figure 2:**
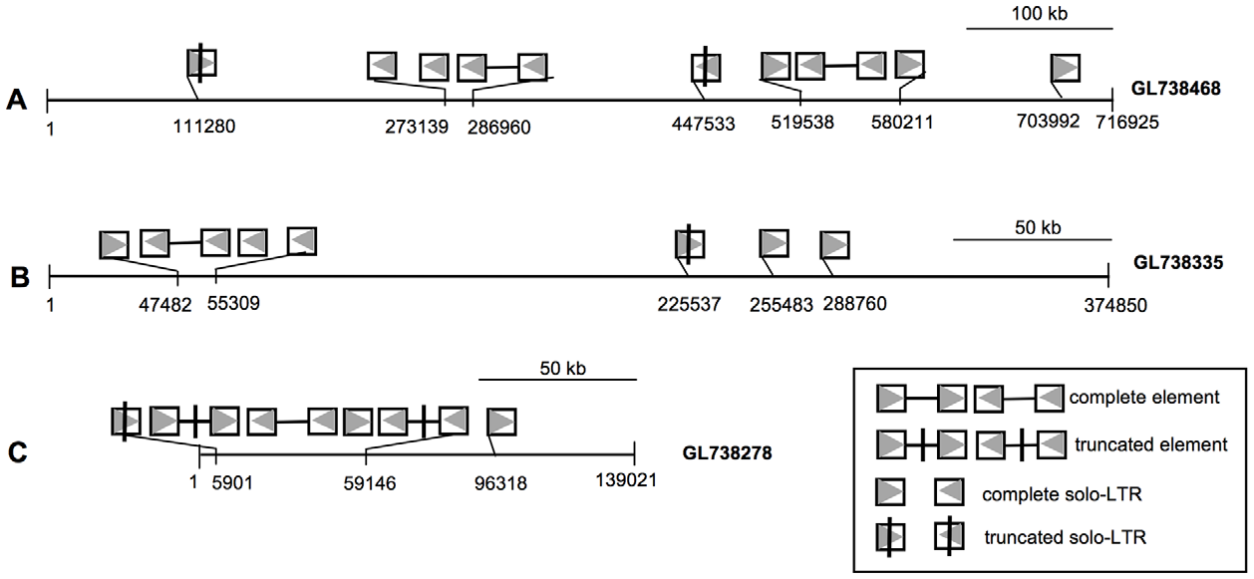
Uneven distribution of PbTRIM elements and their solo-LTRs in the *P. barbatus* genome. PbTRIM elements and solo-LTRs tend to form closely located clusters in some genomic regions.

Elements of the PbTRIM family are distributed unevenly in the host genome. Among all the 897 *P. barbatus* genome scaffolds deposited in GenBank (total size = 230.3 Mb, see [19]), PbTRIM elements and solo-LTRs were found in 152 of them (56.67 Mb in total size), significantly fewer than would be expected if they are distributed randomly in the genome (randomization test, P<0.01). Further sequence analysis revealed that some elements tend to form closely located clusters. For example, a 716.9-kb genomic region was found to contain two complete elements, five solo-LTR and two truncated solo-LTRs, resulting in a transposon density of 12.6 elements per Mb genome sequence (Figure 2A). In a 374.9-Kb and a 139-Kb genome region, PbTRIM elements and solo-LTRs are present in even higher densities (18.7 and 43.2 elements/Mb, respectively; Figure 2B, 2C).

**Figure 3:**
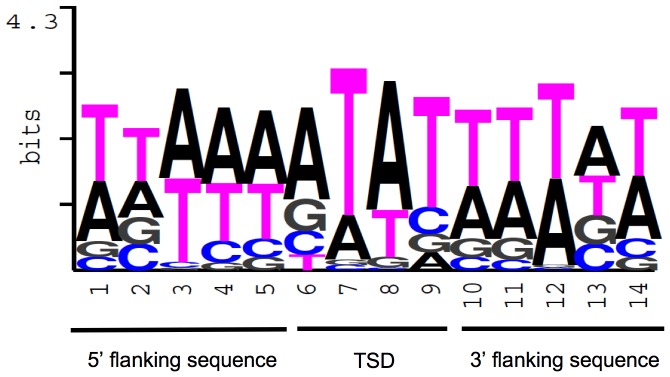
PbTRIM elements preferentially insert into AT-rich region in the *P. barbatus* genome. TSDs along with the flanking sequences from 59 complete PbTRIM elements and 112 solo-LTRs were used to generate the sequence logo.

Many transposons use preferred sites, or “hotspots”, for integration [23]. To examine whether PbTRIM elements show insertion specificity in the host genome, we selected a total of 59 complete elements and 112 solo-LTRs with intact TSDs and flanking sequences, and analyzed the nucleotide composition of the 4-bp TSDs along with the 5-bp flanking sequences on the both ends. Among the 171 analyzed TSDs, the overall GC content of the TSD sequences is 18.1%; the most frequent nucleotide at each of the four positions (5′ – 3′) is A (56.1%), T (74.3%), A (68.4%) and T (63.7%). The 5′ and 3′ flanking sequences are also AT-rich, with the overall GC contents being 19% and 20%, respectively (Figure 3, Table 1). In comparison, GC content of the whole *P. barbatus* genome is 37% [19], which is nearly twice as high as those of the TSDs and their flanking sequences. The results suggest that PbTRIM elements preferentially inserted into AT-rich regions in the host genome.

**Table 1:** Nucleotide frequencies of TSD and its flanking sequences of complete elements (n = 59) and solo-LTRs (n = 112) of PbTRIM family.

Position	−5	−4	−3	−2	−1	T1	T2	T3	T4	+1	+2	+3	+4	+5
A	63	45	84	78	79	96	37	117	17	57	62	78	59	68
C	13	32	5	23	18	25	4	4	26	13	10	4	30	18
G	15	30	3	9	14	35	3	8	19	21	22	4	34	14
T	80	64	79	61	60	15	127	42	109	80	77	85	48	71

Note: T1 and T4 represent the 4 nucleotides of the TSD. The negative (−) and positive (+) signs respectively denote the upstream and downstream position of the nucleotide from the 4-bp TSD region.

### Phylogeny and age of the PbTRIM elements

**Figure 4:**
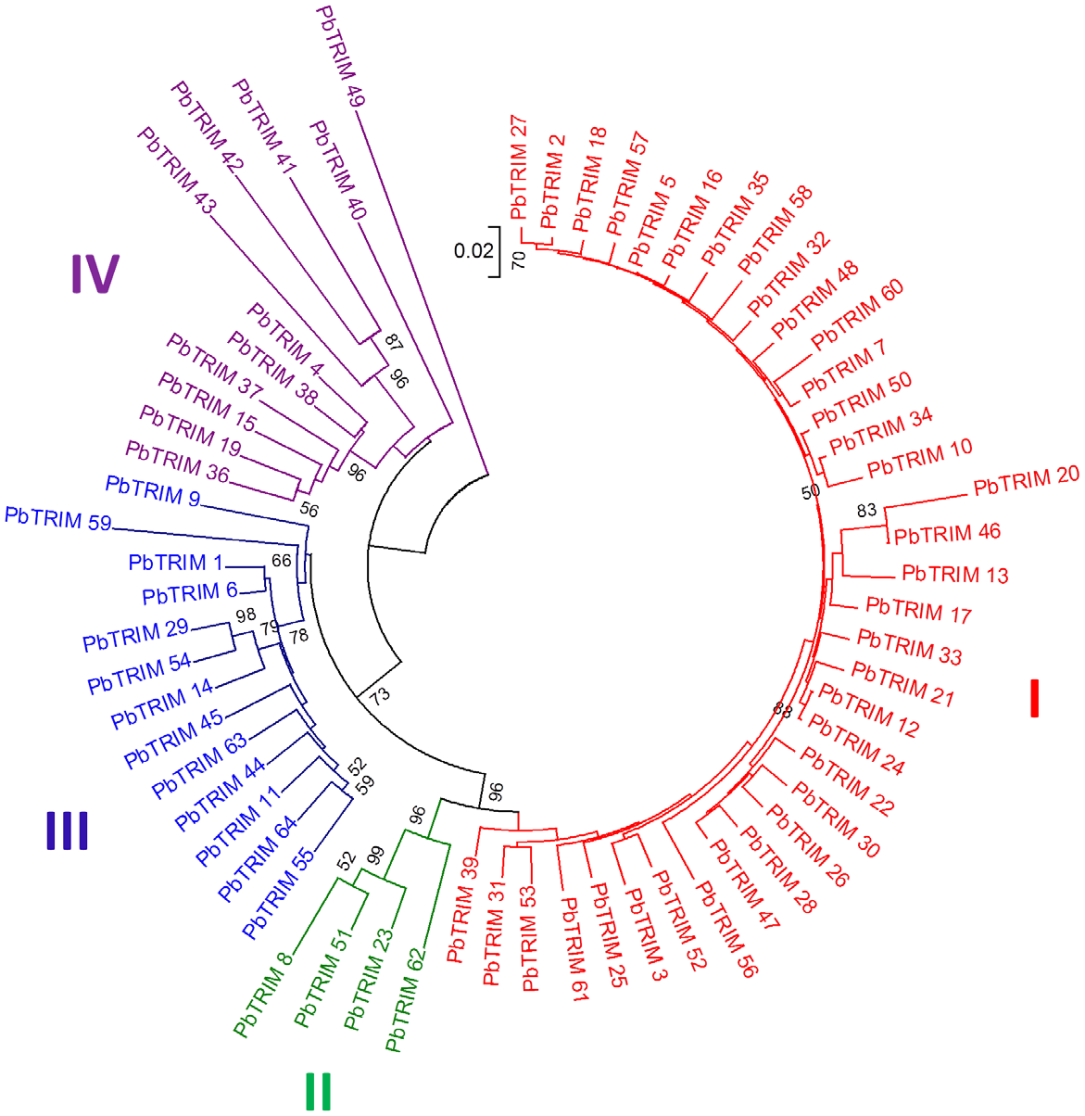
Phylogenetic relationship among the complete PbTRIM elements identified from the *P. barbatus* genome. The neighbor-joining tree was produced with Mega 5.0. The evolutionary distances were computed using the Tamura-Nei method. All branch lengths are drawn to scale. The 64 complete elements included in the analysis can be largely grouped into 4 subfamilies.

To investigate the evolutionary relationships of the PbTRIM elements, we constructed a neighbor-joining phylogenetic tree from aligned nucleotide sequences of 64 complete elements (we removed three elements containing ambiguous bases from the analysis). These elements can be divided into four subfamilies based on their sequence divergence and the phylogenetic tree (Figure 4). Subfamilies I and II form well-supported sister clades. Subfamily I includes the majority of PbTRIM elements (56%) and shares a conserved sequence pattern in the internal region (Figure 1B), while the remaining subfamilies show little conservation in the internal region. Subfamily III forms a moderately well-supported sister clade to subfamilies I and II, which together are sister to subfamily IV (Figure 4). PbTRIM49, which is highly variable in both the LTR and internal regions, did not cluster with any of the subfamily clades; we tentatively group it into subfamily IV since this group contains a couple of highly divergent elements (PbTRIM40-44), as indicated by their long branches.

**Figure 5:**
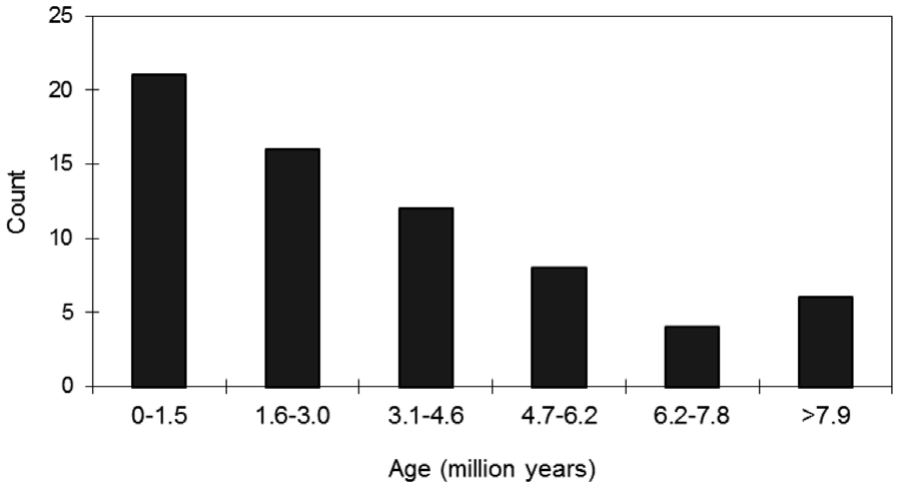
Frequency distribution of the estimated age of 67 complete PbTRIM elements from the *P. barbatus* genome.

The estimated ages of the 67 complete PbTRIM elements range from 0 to 12.9 million years, with most of them (∼85%) being less than 6 million years old (Figure 5). Notably, 10 of the elements have two identical LTRs, resulting in their estimated insertion times to be zero. The data indicates possible recent retrotransposition of these elements. Elements of subfamily I share a high level of sequence similarity and thus have the shortest estimated insertion time (1.7 million years in average) in comparison with other groups. As expected, elements of subfamily IV are most variable in their sequences and represent the oldest group (7.8 million years in average) of TRIM in the host genome. The estimated age of subfamily II and III were intermediate between the other two groups (5.0 and 4.4 million years, respectively).

### Identification of genes associated with PbTRIM

**Figure 6:**
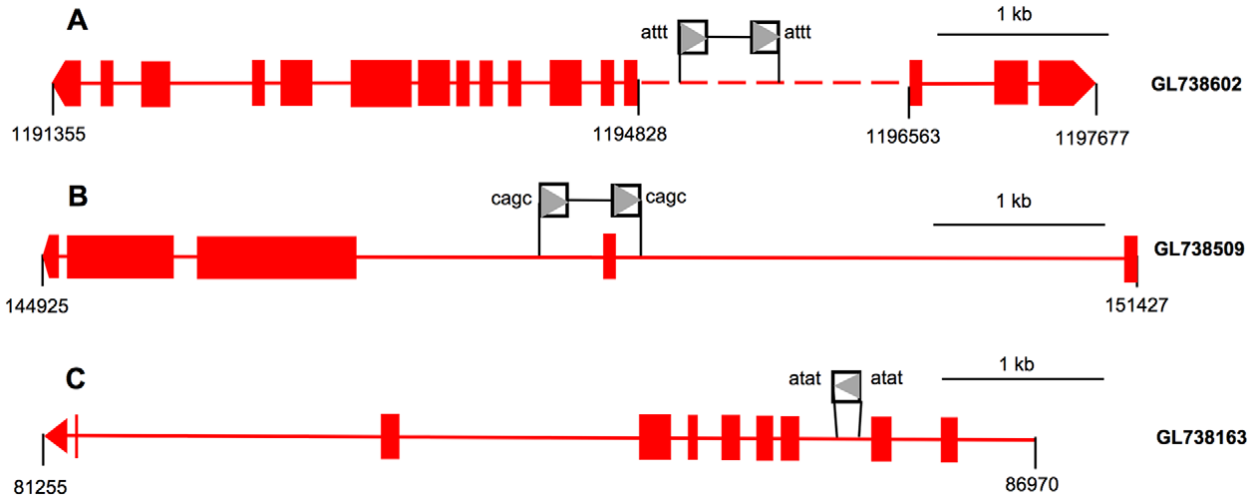
Examples of PbTRIM elements and solo-LTRs located within or near genes. Exons of predicted genes are shown as red boxes, with the last exons denoted as pentagons to indicate the transcription orientation. The solid and the dashed lines represent gene introns and intergenic regions, respectively. The direction of the LTR sequences is indicated by the grey triangles in the open square, and the TSD sequences are shown on the either side of the element or solo-LTR. (A) A complete PbTRIM element is located within 1-kb from the transcription start sites of two genes (upstream: protein SERAC1, downstream: GTP-binding protein ypt7). (B) A complete element contributes sequence to the second exon-intron boundary of the gene encoding SET and MYND domain-containing protein 4. (C) A solo-LTR is located within the first intron of a gene encoding the focal adhesion kinase 1.

**Figure 7:**
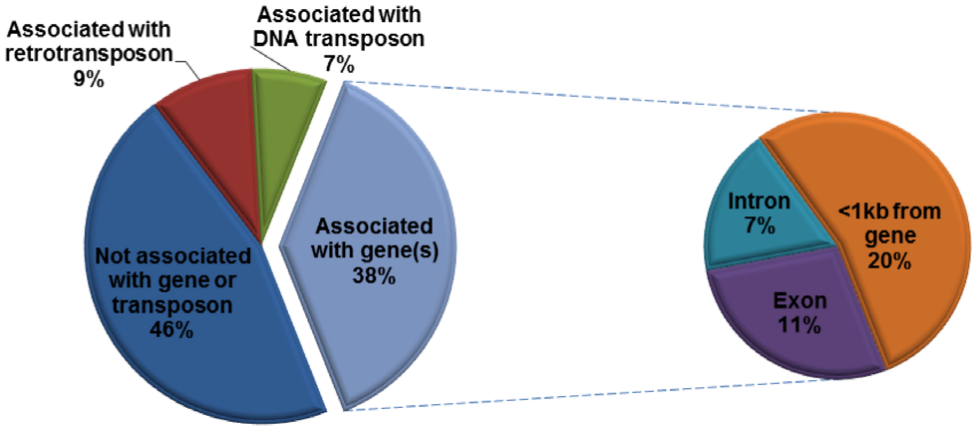
Association of PbTRIM with genes in the *P. barbatus* genome. Among a total of 243 complete elements, solo-LTRs and element fragments of PbTRIM family, 100 (38%) have overlap with gene exons and/or introns, or located within 1-kb from a predicted gene.

Previous studies revealed that plant TRIMs could integrate near or within genes and affect gene structure and function [10]–[11]. To investigate the association of PbTRIM elements with genes, we retrieved 67 complete elements, 153 solo-LTRs and 43 element fragments along with their 10-kb 5′ and 3′ flanking sequences to identify possible coding segments. Thirty-five complete elements (34%), 66 solo-LTRs (41%) and 15 (34%) element fragments were present within 1 kb from predicted genes, or had sequence overlap with gene introns and/or exons (Figure 6 and 7). Among the 157 predicted genes associated with the elements, over 50% have significant BLASTP hits (E-value = 1e-5 or less) with genes deposited in the NCBI nr protein database, 30% were matched to transposable elements (mostly retrotransposon and mariner elements), and the remaining have no significant BLASTP march (Figure 7). A list of the known genes is reported in Table S1. These genes encode a variety of proteins that are involved in a variety of important biological processes, such as transcription, regulation of transcription or translation, signal transduction, cell cycle, DNA repair, protein transport and protein folding.

### Homologous PbTRIM elements in other ant genomes

To investigate whether PbTRIM is present in other ant species, we used the complete PbTRIM elements as queries to search against the Fourmidable ant genomic database [24], the Hymenoptera Genome Database [25], and other insect genomes deposited in the NCBI Whole Genome Shortgun Contigs database. Using a cut-off E-value of 1e-5, significant matches were found from the 6 non-*Pogonomyrmex* ant genomes publically available to date (i.e., *Atta cephalotes*, *Acromyrmex echinatior*, *Camponotus floridanus*, *Harpegnathos saltator*, *Linepithema humile* and *Solenopsis invicta*). However, most of the matches share <70% nucleotide identity with PbTRIM elements along a <50 bp region, and they do not seem to belong to any LTR retrotransposon based on the analysis by the LTR_FINDER program. We found only four exceptions from the genome of the Argentine ant, *L. humile*. Those sequences share ∼70–90% nucleotide similarity with PbTRIM elements over 90–198 bp regions. Among these matched fragments, two are the partial sequences from two complete LTR retrotransposon elements. One of the elements is 657 bp in size and shares 79% identity along 90 bases with PbTRIM elements within the LTR region (GenBank scaffold accession number: ADOQ01017691.1, location 11918–12574); the other one is 1192 bp in length and shares ∼70% nucleotide identity with PbTRIM elements along >80% of their LTR regions (GenBank scaffold accession number: ADOQ01000947.1, location 19148–20339). These two elements share 93% identity in their LTR sequences but little sequence similarity in their internal regions. Also, their internal sequences are very different with those of PbTRIMs. Both of the elements lack coding capability, have the same conserved PBS motif and generate 4-bp TSDs (ATAT and ATTA, respectively) as PbTRIM elements, thus represent homologous PbTRIM elements in *L. humile*.

Using a pair of PCR primers complementary to the conserved LTR region of many PbTRIM elements, successful PCR amplification was produced from genomic DNAs of 8 ant species/lineages, including *P. barbatus*, *P. rugosus*, the *Pogonomyrmex* H and J genetic caste determining lineages, *Pheidole desertorum*, *Pheidole hyatti*, *Lasius alienus* and *Lasius neoniger*. No amplification was obtained from 5 other ant species tested in this study (*Aphaenogaster rudis*, *Camponotus pennsylvanicus*, *Crematogaster sp.*, *Messor pergandei* and *Solenopsis xyloni*. See Table 2 for more information about the examined species).

**Table 2:** Ant species/lineages used for PCR amplification of PbTRIM elements.

Subfamily	Species/lineages	Collection Locale
Myrmicinae	*Pogonomyrmex barbatus*	San Patricio Co., TX
	*Pogonomyrmex rugosus*	Cochise Co., AZ
	*Pogonomyrmex H lineage* *	Sierra Co., NM
	*Pogonomyrmex J lineage* *	Cochise Co., AZ
	*Pheidole desertorum*	Hidalgo Co., NM
	*Pheidole hyatti*	Hidalgo Co., NM
	*Solenopsis xyloni*	Hidalgo Co., TX
	*Messor pergandei*	Pima Co., AZ
	*Crematogaster sp.*	Dallas Co., TX
	*Aphaenogaster rudis*	Hampden Co., MA
Formicinae	*Lasius alienus*	Chittenden Co., VT
	*Lasius neoniger*	Chittenden Co., VT
	*Camponotus pennsylvanicus*	Chittenden Co., VT

* *Pogonomyrmex* H and J lineages are populations of *Pogonomyrmex barbatus* x *Pogonomyrmex rugosus* hybrid ancestry. See Method section for more details about the two lineages.

**Figure 8:**
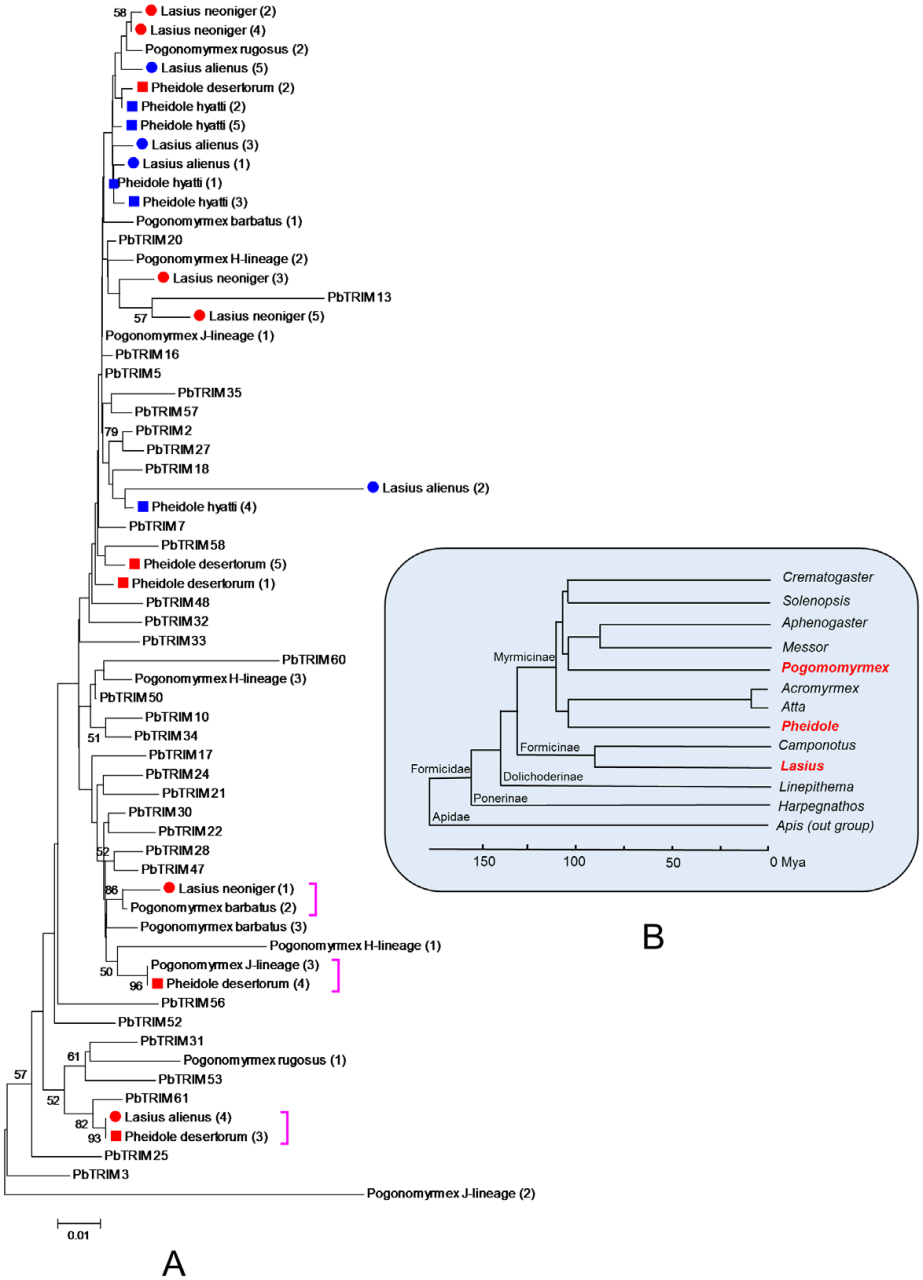
Phylogenetic relationships of homologous PbTRIM sequences and their host species. (A) Neighbor-joining phylogenetic tree of homologous PbTRIM sequences derived from different ant species/lineages. The tree was produced from 36 PbTRIM elements (all belong to subfamily I) originally identified from the *P. barbatus* genome and 31 partial sequences identified from different ant species/lineages through PCR amplification. Branch lengths are proportional to the genetic distances. Only bootstrap values greater than 50 are shown. Homologous sequences derived from non-*Pogonomyrmes* ant species/lineages were indicated by different colored shapes. Numbers in brackets denote the clone numbers of individual sequences. Close square brackets indicate elements from different ant species that share high sequence similarity (see text for more details). (B) Phylogenetic relationship of 12 ant genera examined in this study. The tree was generated following Moreau *et al.* (2006) [41] and Brady (2006) [47], with *Apis* included as an out-group. PbTRIM homologues were found in three of the genera (shown in red) through PCR analysis.

Sequencing analysis on the recovered PCR products confirmed successful amplification of the targeted DNA fragments. A total of 31 non-redundant DNA fragments (356 to 445 bp in size) were obtained from the 8 ant species/lineages (GenBank accession numbers JQ356726–JQ356756). We further analyzed the phylogenetic relationship of these homologous PbTRIM sequences and 63 PbTRIM elements originally identified from the *P. bartbatus* genome (PbTRIM46 was removed from the analysis due to a large deletion in its target region). Interestingly, all the PCR-amplified homologues from non-*Pogonomyrmex* species were clustered into PbTRIM subfamily I. To improve the resolution of the phylogeny, we reanalyzed only subfamily I PbTRIMs. The neighbor-joining tree shows a discordant pattern between the evolutionary relationship of the elements and their hosts (Figure 8A). Although the three ant genera in which elements were discovered fall into two well-supported subfamilies, and all diverged from one another over 100 million years ago (mya), the neighbor-joining tree of PbTRIM subfamily I showed virtually no resolution outside of the tips of the tree. Of the three terminal clusters with strong statistical support (>80%), all grouped pairs of elements derived from different genera or even subfamilies (Figure 8A). PbTRIM and their homologues also showed a patchy distribution pattern among the 12 ant genera in which the presence or absence of PbTRIM was examined. For example, for the 7 genera of Myrmicinae, PbTRIM was detected in *Pogonomyrmex* and *Pheidole* but not in the others (Figure 8B).

To further examine the evolutionary history of PbTRIM in ant species, ancestral states of the presence or absence of the retroelement were reconstructed with MacClade program [26], based on a phylogenetic tree including 13 species that have proved to either have (7 ant species) or lack (5 ant species and the outgroup *Apis mellifera*) PbTRIM. This analysis indicated multiple gains and/or losses of the element within the family Formicidae. Three most parsimonious reconstructions (MPRs) of ancestral states were generated (Figure S1). These reconstructions all require 4 steps with 1–4 gains and 0–3 losses of the trait. ACCTRAN tracing, which favors change as close as possible to the root, supports a single gain of the retroelement with 3 secondary losses; DELTRAN tracing, which favors change as far as possible to the root, supports 4 parallel gains of the retroelement.

## Discussion

As a unique group of LTR retrotransposon characterized in plants, TRIMs are distinguished by their exceptionally small overall size and non-autonomous internal domain. TRIMs are abundant in the host genome, and elements from different species share sequence similarity, especially in the LTRs [10], [14]. Likewise, PbTRIM elements from the red harvester ant have similar size and structure. Multiple copies of PbTRIM elements are present in different locations of the *P. barbatus* genome, and highly similar PbTRIM sequences were detected from different ant species. The ant PbTRIM family, however, are apparently different from plant TRIMs in two respects. First, PbTRIM apparently originated independently, as elements share no overall sequence similarity with plant TRIMs, including the PBS motif, which is conserved in many LTR retrotransposon families [27]. During replication of the retroelement, the PBS provides a binding site for a certain cellular tRNA that acts as a primer for reverse transcription [27]–[28]. Plant TRIMs have a PBS motif (12–18 nt) complementary to methionine tRNA, whereas the PBS of PbTRIM family (∼15 nt) matches leucine tRNA of *D. melanogaster*. Second, plant TRIMs are flanked by 5-bp TSDs without apparent sequence pattern [10], [13], while PbTRIM elements generate 4-bp TSDs with the most common sequence being ATAT.

Like TRIM elements in plants, several observations suggest that PbTRIM is probably transpositionally active in the host genome. 1) Elements are generally young based on the nucleotide divergence between the two LTR regions of the retrotransposons. The two LTR sequences of many PbTRIM elements are nearly identical, suggesting that PbTRIM elements were recently, and are perhaps still, active; 2) Many PbTRIM elements at different genomic locations share high overall sequence similarity, indicating that they may be the products of recent transposition events; and 3) Target site duplications (TSDs) have been found for most of the complete elements and solo-LTRs. Retrotransposon integrases create staggered cuts at the target sites, resulting in TSDs as they insert new elements. Therefore, detection of TSDs flanking genomic retroelement copies provides evidence for retrotransposition [11], [29]. The possible mobility of PbTRIMs thus raises the question of how PbTRIMs move around in the host genome, given that the elements lack all the coding domains (in particularly *gag* and *pol* genes) necessary for retrotransposition. Non-functional defective TEs can often “borrow” the enzymatic machinery from the full-length functional elements of the same family. So far we haven't found any functional PbTRIM element, or any other autonomous TE that shows sequence similarity with the family. A similarly situation exists for plants TRIMs. Although elements have shown to be actively involved in reconstructing plant genes, the autonomous TRIM has not yet been discovered. It has been proposed that TRIMs in plant are probably cross-activated in trans by autonomous partners from other TE families [10]. It has been well documented that non-autonomous TEs, such as miniature inverted repeat transposable elements (MITEs) in some species, can be activated by autonomous partners (*Tcl-Mariner* or *PIF-Harbinger* TEs) that share little sequences similarity with them [3]. Similar mechanism may operate in PbTRIMs.

Like their counterpart in plants, PbTRIM elements have the potential to affect the structure of host genes. Among all the 264 PbTRIM elements/solo-LTRs/element fragments analyzed in the *P. barbatus* genome, 38% were located near or within genes. The direct insertion of the retrotransposons may cause gene mutation or alter regulation for the affected locus. Moreover, PbTRIM has the potential to affect host genes and genomes by recombinational activities. Retroelements can act as agents of genome restructuring primarily by providing sources of homology for unequal recombination. In yeast, unequal recombination between directly repeated Ty retroelements at adjacent sites can result in reciprocal duplications and deletions of the DNA between the two elements, while unequal exchange between elements in opposite orientations will yield an inversion of the DNA between the elements. Similar ectopic (unequal) exchange between elements on different chromosomes can give rise to reciprocal translocation [30]–[31]. In *Drosophila*, several different transposable elements, including two retrotransposons *HeT-A* and *TART*, are involved in chromosome rearrangements. The process seems to involve ectopic recombination between elements inserted at different chromosomal sites [32]. Solo-LTRs are a common outcome of unequal homologous recombination between the two directly repeated LTRs of a single element [33]. In addition to 67 intact PbTRIM elements, we identified 159 solo-LTRs in the *P. barbatus* genome, suggesting occurrence of many unequal recombination events.

One unique feature of PbTRIM, which has not been characterized in plant TRIMs, is that elements preferentially insert into AT-rich genomic regions. Unlike the genomes of many other species, where GC-rich regions tend to have more genes, in the genomes of *P. barbatus* and other Hymenopteran species sequenced to date, genes tend to occur in AT-rich regions [20]. Given the fact that 38% of the PbTRIM elements/solo-LTRs are found near or within genes, it is possible that preferentially inserting into AT-rich sites has provided PbTRIM elements relatively more chances to integrate into the gene-rich, transcriptionally active part of the genome, thus gaining opportunities for further transcription and transposition. Meanwhile, relative to other large LTR retrotransposons, the small size makes members of the family less likely to cause severe mutations within gene-rich regions. A preference for AT-rich sequences has also been reported for some LTR retrotransposons in *Drosophila* [34]–[35]. Although the underlying mechanisms are not clear, previous data from other transposable elements suggest several possibilities. It has been found that *Tc1*/*mariner*-type DNA transposons specifically target AT-repeats for insertion. However, target site selection is not determined by specific primary sequence, but at the level of DNA structure, including bendability, A-philicity and a symmetrical pattern of hydrogen bonding sites in the major groove of the target DNA [36]. Similarly, a recent study revealed that in *D. melanogaster*, all the *gypsy* group LTR-retrotransposons with three ORFs and their derivatives with two ORFs show certain target site specificity. The integration specificity is associated with the structural features of the target DNA (DNA-bending, A-philicity, proteininduced deformability) and the integrase (such as chromodomain) [37]. In yeast, retrotransposon *Tf1* frequently integrates into the promoters of RNA polymerase II (Pol II) transcribed genes [38], and the integration pattern of *Tf1* in the *fbp1* promoter is determined directly by one of the activators, *Atf1p* [39]. LTR retrotransposons *Ty1* and *Ty3* specifically insert into the initiation sites of genes transcribed by RNA polymerase III (Pol III) [40], whereas *Ty5* integrate preferentially within domains of heterochromatin, and the specific target site is determined by interactions between an amino acid sequence motif at the C terminus of *Ty5* integrase and a heterochromatin protein [41]. Therefore, it is possible that the integration “hotspots” for PbTRIM family are not defined by primary sequence preferences. A comprehensive understanding of the integration specificity of the family requires further study on the secondary structure of the insertion site, more knowledge about it autonomous counterpart and transposition mechanism.

In plants, TRIM is a fairly abundant family that has been found in all vascular species investigated to date. Their origin has been placed at least in the Permian, 250 Million years ago [11]. Among the ant species examined in this study, the most ancestral taxon in which homologous PbTRIM elements were detected is the Argentine ant *L. humile*, a species in the subfamily of Dolichoderinae, which probably diverged from the other groups at least 70 million years ago [42]–[43]. However, it is uncertain whether PbTRIM is as ancient in ant species as the TRIM families in plants. Using the 12 ant genera in which the presence or absence of PbTRIM is known, reconstruction of ancestral character states produced three equally most-parsimonious models explaining the presence/absence pattern of the retroelement (Figure S1). The ambiguous reconstructions could not be resolved by ACCTRAN and DELTRAN optimizations: while the ACCTRAN tracing supported the hypothesis that PbTRIM first appeared in *L. humile* and was secondarily lost 3 times thereafter, DELTRAN favored 4 independent gain events along the tree.

On the other hand, relationships among elements in subfamily I suggest an important role of horizontal transfer in the current distribution of PbTRIM across ant taxa. The short estimated insertion times and high copy number within this subfamily indicate that this is a young and transpositionally active group; this is further supported by the shared structural patterns in the non-coding internal region, which is not expected to be under purifying selection and rapidly degrades over time as evidenced by the loss of discernible patterns in the older subfamilies (II–IV). Despite their young age, however, elements with high sequence similarity in both the LTR and internal regions were found in distantly related ant species (Figure 8A). For example, a PbTRIM amplicon from *Pogonomyrmex* J-lineage is 100% identical with an amplicon from *Pheidole desertorum*; two amplicons from *L. neoniger* and *P. barbatus*, which are in different subfamilies that split more than 100 million years ago [42]–[43], share 99% sequence similarity with one another. Although we cannot completely rule out alternative hypotheses, such as differential fixation of ancestral polymorphisms, differential evolutionary rates or selective constraints [44], the most likely explanation for such high sequence identity between the TEs is recent horizontal transfer. An increasing amount of evidence has accumulated over the years that transposable elements can move horizontally in eukaryotes, often at considerable frequency [45]. More knowledge on the biology and distribution of PbTRIM in other ant species is necessary to verify our inference of horizontal transfer of PbTRIM elements, and to better understand the origination and evolution of this retroelement.

## Experimental Procedures

### Ethics Statement

No specific permits were required for our field collection/activities. The locations are not privately-owned or protected, and the ant species collected are not endangered or protected.

### Annotation of LTR retrotransposons

The draft genome assembly of *P. barbatus* (Version 3) was downloaded from the NCBI website (http://www.ncbi.nlm.nih.gov) and scanned for LTR retrotransposons with the LTR_FINDER program [22]. The default setting was used with the following exceptions: both the minimum LTR size and the minimum distance of LTRs (internal domain) were set at 50 bp, and the *D. melanogaster* tRNA database (Release 5 April 2006) was used to predict the primer binding site (PBS). The output sequences were manually checked to confirm the boundary of the LTRs and target site duplicates (TSD). To detect the elements that could have been missed by the LTR-FINDER software, annotated complete elements were used for BLASTN searches against the *P. barbatus* genome. Finally, an all-against-all sequence alignment was performed to group the identified complete elements into different subfamilies.

### Estimation of insertion time

The insertion time of an LTR retrotransposon can be estimated by the nucleotide divergence between its 5′ and 3′ LTR sequences [46]. This method is based on the assumption that the sequences of the two LTRs were identical at the time of integration and accumulated point mutations independently with time. Therefore, the nucleotide substitution rate between the two LTRs reflects the time since the insertion event. The MEGA5 program [47] was used to calculate the number of substitution mutations using the Kimura-2 parameter method. The insertion time of retrotransposons was estimated with the formula T = K/2r, where T, K and r are time of divergence, average number of substitutions per aligned site and an average synonymous substitution rate, respectively. Since synonymous substitution rates of ant nuclear genes have not yet been examined, we estimated the rate based on pairwise analysis of 95 orthologous protein-coding sequences from three ant species, *Camponotus floridanus*, *P. barbatus* and *Solenopsis invicta* (data not shown). In comparison, the average rate we estimated for ant species (0.54×10^−8^ synonymous substitutions per site per year) is about 1/3 of that has been estimated for Drosophila (1.6×10^−8^), which was calculated from 39 genes between *D. melanogaster* and *D. obscura* groups [48] and have been used to calculate the age of retrotransposons in *D. melanogaster* [38].

### Construction of sequence Logos

To examine the nucleotide composition of the insertion site of PbTRIM elements, we analyzed TSDs and their 5-bp 5′ and 3′ flanking sequences for a total of 59 complete PbTRIM elements and 112 solo-LTRs. Commonalities and differences within multiple aligned sequences were represented by a sequence logo generated with CheckAlign 2.0 (http://gydb.org/index.php/Checkalign_sequence_logos).

### Phylogenetic analysis

Sequences of PbTRIM elements were aligned using ClustalW (http://www.ebi.ac.uk/Tools/msa/clustalw2) under default settings. The aligned dataset was subjected to neighbor joining analysis based on the Tamura-Nei (1993) model with MEGA 5 [47]. Mutation rates among sites were treated as uniform, and all positions containing alignment gaps and missing data were eliminated only in pairwise sequence comparisons (pairwise deletion option). The bootstrap values for nodes were computed from 1000 replicates.

To examine the evolutionary history of PbTRIM in ants, we built a phylogenetic tree for 12 ant species following Moreau *et al.* (2006) [42] and Brady (2006) [43], using *Apis mellifera* as the outgroup. Among these ant species, 7 have PbTRIM elements present in their genome sequences or detected by PCR amplification; the remaining 5 species and *A. mellifera* lack the retroelement in their sequenced genomes. Although our PCR analysis did not detect PbTRIM elements in some other ant species, considering that possible homologous PbTRIM elements could be missed by PCR when there were mutations within the primer annealing regions, we excluded them from the phylogenetic analysis. Based on the phylogenetic tree, MacClade program 4.06 [26] was used to reconstruct ancestral states (presence or absence) of the retroelement. Standard parsimony method as well as DELTRAN and ACCTRAN methods was applied for the analysis.

### Randomization analysis of the genomic distribution of PbTRIM

Using a custom python script, we performed a randomization analysis to test whether the retroelements are distributed randomly in the host genome. In the analysis, a total of 270 PbTRIM elements/solo-LTRs were individually re-assigned to a randomly selected position in the *P. barbatus* genome, and the total number of the unique genome scaffolds receiving the elements was calculated. The process was repeated for 5000 times. The observed number of the scaffolds containing PbTRIM elements (n  = 152) was then compared with the computer generated null distribution of scaffold numbers. If the observed scaffold number is smaller than 5% (P-value<0.05) or 1% (P-value<0.01) of the computer generated distribution, we reject the hypothesis that the elements are distributed randomly throughout the genome.

### Association of PbTRIM with genes

We retrieved the upstream and downstream 10-kb sequences immediately flanking the complete PbTRIM elements, solo-LTRs or element fragments from the *P. barbatus* genome assembly. Elements along with their flanking sequences were submitted to FGENESH program (Softberry, Inc., http://linux1.softberry.com/berry.phtml?topic=fgenesh&group=programs&subgroup=gfind) for gene prediction. The identity of the predicted coding segments (CDS) was analyzed by comparing the predicted protein sequences with the NCBI nonredundant (nr) protein database using BLASTP.

### Ant collection, PCR amplification and sequencing analysis

To examine whether homologous PbTRIM elements are present in different ant species/populations, we collected individual workers from one *P. barbatus* population, one *P. rugosus* population and two *Pogonomyrmex* lineages (J and H lineage) of *P. barbatus*/*P. rugosus* ancestry. Both lineages differ with their parental species in showing a strong correlation between female genotype and reproductive caste, a pattern known generally as genetic caste determination (GCD) [16]–[17]. In addition, we collected workers from nine other ant species from different field sites (see Table 2 for more details about the subfamily, name and collection site of each species/lineage).

Depending on the body size of the ant species, DNA was extracted from three legs or the whole body of individual workers using 5% chelex solution, as described by Helms Cahan *et al.* [49]. We designed a pair of PCR primers (Forward: 5′-AATCCAGTCGGTCTCCGTTA-3′; reverse: 5′-ACGACGGCTCCTCTCTGTTA-3′) based on the highly conserved LTR regions in a multiple sequence alignment of PbTRIM elements originally identified from the *P. barbatus* genome. PCR amplification was performed in 20 µl mixtures containing 10–20 ng DNA, 20 mM Tris-HCl (pH 8.4), 2 mM MgCl_2_, 50 mM KCl, 0.2 µM each primer, 200 µM each dNTP, and 1 U Taq polymerase (Invitrogen, Carlsbad, CA, USA). Amplifications were run in Eppendorf Mastercycler epgradient (Eppendorf, Hamburg, Germany) with an initial denaturing step of 94°C for 4 min followed by 34 cycles of 30 s at 94°C, 30 s at 54°C, 1 min at 72°C, and a final extension at 72°C for 5 min.

Amplified products were run on 1% agarose gel and purified with the MinElute Gel Extraction Kit (QIAGEN, Valencia, CA, USA). The purified PCR products were cloned into pGEM-T easy vector system (Promega, Madison, WI, USA). Three to six recombinants from each ant species/lineages were randomly selected for sequencing analysis on the ABI 3730xl DNA Analyzer.

## Supporting Information

Figure S1 **Parsimonious recontructions for the evolutionary history of PbTRIM family in 12 ant species.** Three equally most-parsimonious trees were produced by the standard parsimony method in MacClade program (v4.06). The most parsimonious ancestral states are shown on each branch, with yellow and blue respectively indicate absence and presence of PbTRIM elements. Figure A and C are supported under DELTRAN and ACCTRAN option, respectively. Based on the reconstructions, the evolutionary history of PbTRIM could be equally explained by four gain events (A), two gain and two loss events (B), or one gain and three loss events (C).
(TIF)

File S1 **Multiple alignment of 64 complete PbTRIM elements identified from the sequenced genome of** ***P. barbatus*** **.**
(FAS)

Table S1 **A list of the predicted coding segments (CDS) that have significant BLASTP matches (E-value = 1e-5 or less) to the characterized genes in the NCBI nr protein database.** These predicted CSDs either contain sequences of PbTRIM elements/solo-LTRs, or are located within 1 kb from the latter.
(XLSX)
