# Supplementary figures and images for: A Novel Family of Terminal-Repeat Retrotransposon in Miniature (TRIM) in the Genome of the Red Harvester Ant, Pogonomyrmex barbatus
Source: PLoS One. 2012 Dec 28;7(12):e53401. doi: 10.1371/journal.pone.0053401 (PMC3532108; doi:10.1371/journal.pone.0053401)

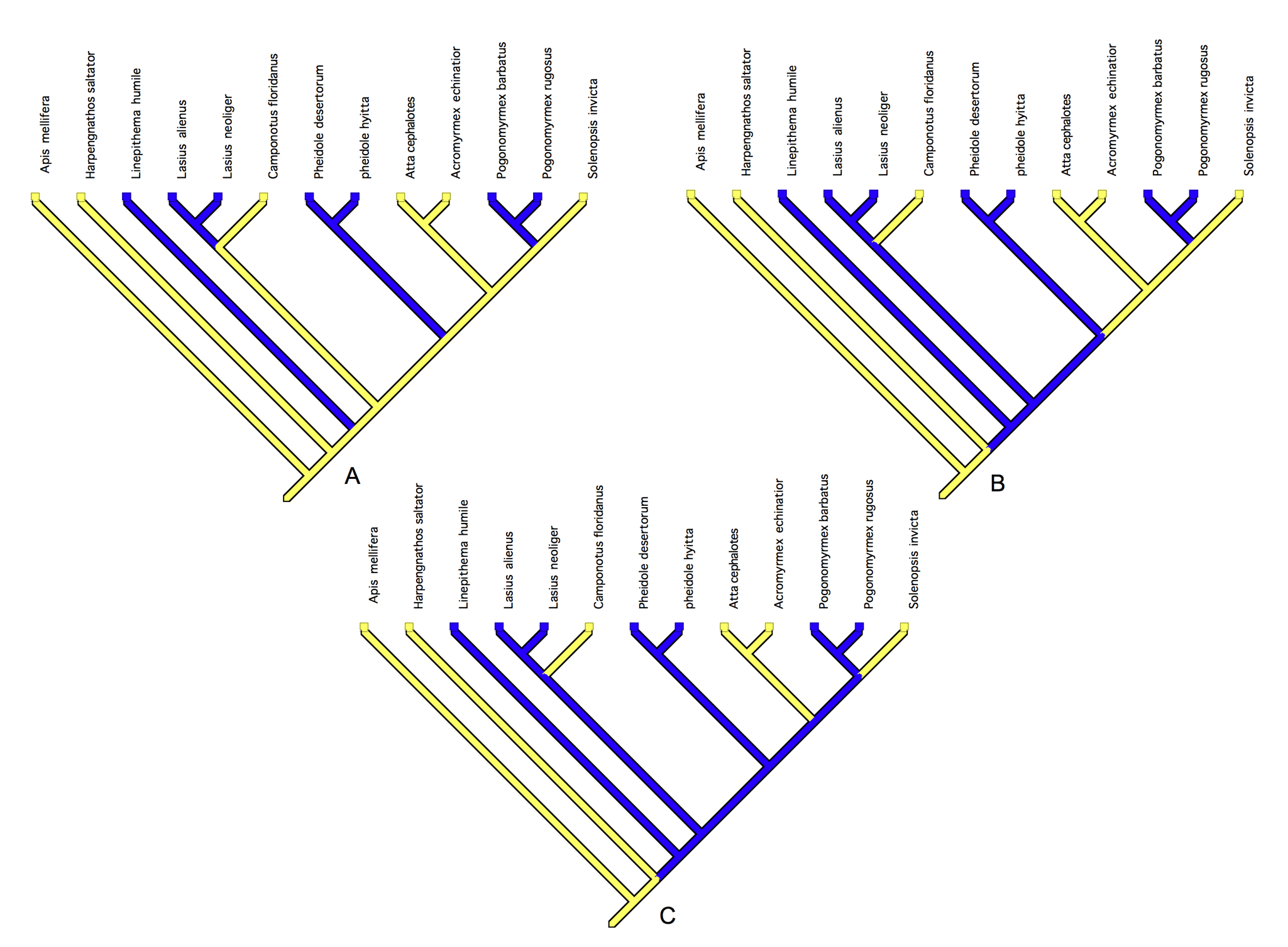

Supplement: Figure S1 — Parsimonious recontructions for the evolutionary history of PbTRIM family in 12 ant species. Three equally most-parsimonious trees were produced by the standard parsimony method in MacClade program (v4.06). The most parsimonious ancestral states are shown on each branch, with yellow and blue respectively indicate absence and presence of PbTRIM elements. Figure A and C are supported under DELTRAN and ACCTRAN option, respectively. Based on the reconstructions, the evolutionary history of PbTRIM could be equally explained by four gain events (A), two gain and two loss events (B), or one gain and three loss events (C). (TIF) [file pone.0053401.s001.tif]
